# Effectiveness of omega-3 poly unsaturated fatty acids on the treatment of gingival hyperpigmentation: a randomized controlled clinical trial

**DOI:** 10.1186/s12903-026-08354-y

**Published:** 2026-04-28

**Authors:** Reda Elsayed Hussien, Rehab Salah Eldin Mahmoud, Shaimaa Mohammed Morsy

**Affiliations:** Department of Oral Medicine & Periodontology, Faculty of Dentistry, Suez Canal University, Ismailia, Egypt

**Keywords:** Mesotherapy, Polyunsaturated fatty acids (PUFAs), Gingival hyperpigmentation, Omega-3, Non-invasive treatment, Aesthetic dentistry

## Abstract

**Background:**

In dentistry, gingival hyperpigmentation is a common aesthetic issue caused mainly by excessive melanin deposits. Traditional surgical methods for depigmentation, like surgical techniques, can be effective, but they often associated with pain after the procedure and longer healing times. Because of their anti-inflammatory and antioxidant qualities, omega-3 polyunsaturated fatty acids (PUFAs) have demonstrated potential benefits in tissue regeneration and dermatological depigmentation.

**Objective:**

To investigate the effectiveness of locally and systemically administered Omega-3 (PUFAs) in reducing gingival pigmentation, thereby contributing to advancements in aesthetic periodontal care.

**Methodology:**

Two groups were randomly selected from thirty patients who presented with mild to moderate gingival pigmentation grade (1,2 or 3) in the maxillary esthetic area. Group I was given a daily oral dose of 2 g of Omega-3 PUFAs before and after one weekly intragingival injections of 1–2 g of omega-3. Group II was depigmented using a traditional surgical technique. At one, three, and six months, the primary outcome Dummett Oral Pigmentation Index (DOPI). Secondary outcomes Hedin index measured at 1,3,6 months, Visual Analogue Scale (VAS) and wound healing were measured at one and seven days following the procedure. Hemorrhage during the surgery were also noted.

**Results:**

Both groups displayed a significant decrease (*P* ≤ 0.05) in the DOPI and Hedin index at various time points. However, after six months, there was no significant difference between the groups (*P* ≥ 0.05). When compared to Group II at various time points, group I displayed a statistically significant decrease (*P* ≤ 0.05) in VAS, hemorrhage, and wound healing. gingival pigmentation.

**Conclusion:**

Omega-3 PUFAs oral mesotherapy is a minimally invasive, safe, and aesthetically acceptable alternative to surgical depigmentation. Its antimelanognic activity makes it a good option for gingival hyperpigmentation treatment.

**Trial registration:**

This research was officially registered in clinicaltrial.gov (NCT07067515) on (15/7/2025).

## Background

Perioaesthetics are now highly demanded, as they not only address biological and functional issues but also create harmony between the white and pink components (teeth and gingiva). A characteristic macroanatomical aspect of healthy gingiva is its pink colour, which varies in intensity based on factors like keratinization level, gingiva thickness, vascularization level, hemoglobin reduction, and melanocytic cell presence [1, 2].

The excessive accumulation of melanin granules in melanocytes in the basal and suprabasal layers of the epithelium results in gingival hyperpigmentation (GHP) [3]. Numerous internal or external factors might lead to hyperpigmented gingiva. The most common cause of gingival hyperpigmentation is physiologic pigmentation, which is described as the most prevalent multifocal or diffuse oral mucosal pigmentation, predominantly found in dark-skin populations [4]. The quantity and activity of melanosomes influence the degree of GHP [5] .

Melanogenesis occurs within melanosomes and involves the enzymatic oxidation of tyrosine, producing eumelanin (black brown) and pheomelanin (yellow red) [6, 7]. Tyrosinase and related proteins (TRP-1, TRP-2) control the ratio of these pigments. Melanin is transferred to keratinocytes via cytophagocytosis and exocytosis [8, 9]. Although benign, GHP can pose significant aesthetic concerns, particularly for patients with high smile lines or short upper lips [1] .

Multiple techniques have been proposed for the treatment of GHP, including surgical removal with a scalpel or bur, electrosurgery, cryosurgery, radiosurgery, and laser therapy, as well as nonsurgical approaches such as chemical cauterization [10, 11] and microneedling [12]. While effective, these methods are often invasive, costly, and associated with postoperative discomfort and recurrence.

Gingival depigmentation with minimal invasiveness has been proposed with mesotherapy technique that involve delivery of depigmented agent directly into the region between the basement membrane and the superficial connective tissue [13].

Polyunsaturated fatty acids (PUFAs) in particular have become recognized as bioactive substances with potential for therapeutic use. Essential macronutrients, fatty acids contribute to cellular signaling, membrane structure, and energy metabolism [14, 15].

There is substantive evidence to support the regular consumption of n-3 PUFA, as these are beneficial for growth, development, health and the welfare of humans and animals [16]. The two families of PUFAs are Omega-3 (n-3) and Omega-6 (n-6). Omega-3 fatty acids, especially Docosahexaenoic Acid (DHA) and Eicosapentaenoic Acid (EPA), have important functions for anti-inflammatory signaling and neuroprotection [9, 17, 18]. 

As it competes with arachidonic acid (AA) for the production of eicosanoids, omega-3 PUFAs decrease inflammation. While AA-derived mediators like Prostaglandin E2(PGE2) and Leukotriene B4 (LTB4) promote inflammation, EPA and DHA produce less potent eicosanoids (PGE3, LTB5) and specialized pro-resolving mediators such as resolvin and protectin [19]. These properties make Omega-3 attractive candidates for managing inflammatory and pigmentary disorders.

The effects of various fatty acids in melanogenesis have been studied with conflicting results [20]. Research has shown that DHA can inhibit Alpha Melanocyte Stimulating Hormone (α-MSH)-induced melanin production in B16F10 melanoma cells by speeding up tyrosinase degradation through the ubiquitin proteasome pathway, without modifying the release of Microphthalmia-associated transcription factor (MITF) [21–23]. Additionally, alpha-linolenic acid (ALA) and linoleic acid (LA) have shown skin-whitening effects through tyrosinase (TYR) inhibition [20, 24] .

Given the dual anti-inflammatory and anti-melanogenic effect of Omega-3 PUFAs, their application in managing GHP presents a promising minimally invasive alternative to conventional surgical techniques. The study intends to investigate the effectiveness of Omega-3 PUFA treatment administered both locally and systemically in reducing gingival pigmentation, thereby contributing to advancements in aesthetic periodontal care.

## Subjects and methods

### Ethical approval

This study was conducted as a randomized clinical trial in accordance with the ethical standards of the institutional research committee and with the Declaration of Helsinki (2013) and was reported in accordance with the CONSORT guidelines for reporting randomized clinical trials (2010). Ethical approval was obtained from the Research Ethics Committee (REC) of the Faculty of Dentistry, Suez Canal University (Approval No.: 784/2024). The study was registered at ClinicalTrials.gov (Registration No.: NCT07067515). Written informed consent was obtained from all participants prior to their inclusion in the study.

### Study sample and sample size calculation

The number of participants needed to find a significant difference in postoperative pigmentation intensity between two treatment groups was estimated by G*Power software (version 3.1.9.4). Based on a study of Chaudhary et al. (2023) [25] the minimum sample size required was 24 patients, with 12 in each group. This was based on an effect size of 1.61, an alpha level of 0.05, and a power of 0.95. However, the final sample size was increased to 30 patients, evenly divided between the two groups, to improve statistical strength and to consider possible dropouts.

### Study setting and patient selection

#### Recruitment

Participants were selected from Suez Canal University’s Oral Medicine and Periodontology Department’s Outpatient Clinic. We maintained strict ethical standards for patient confidentiality and well-being. Each patient gave informed consent after discussing the study procedures and follow-up schedule in detail.

#### Inclusion and exclusion criteria

Participants were systemically healthy adults aged 21–50 years with mild to moderate physiologic GHP located in the maxillary aesthetic zone, specifically from the canine/first premolar region extending to the corresponding contralateral teeth, assessed using the Dummett and Hedin index (scores 1–3). All participants maintained good oral hygiene and agreed to undergo the minor procedure.

##### Exclusion criteria

Included severe gingival pigmentation, pathological etiology of GHP, any systemic health condition that could interfere with participation or healing, current or previous chemotherapy, smoking, pregnancy, or any other condition deemed unsuitable for study inclusion. Also, participants with liver disease, fatty liver, or other conditions that could be adversely affected by vitamin A intake was excluded.

#### Randomization

Participants were randomly allocated into two groups using a simple randomization (lottery) method. A random allocation sequence was prepared by an independent staff member not involved in patient recruitment or treatment. Numbers from 1 to 30 were written on identical opaque folded paper slips and thoroughly mixed in a closed container. After enrollment, each participant drew one slip, and group assignment was determined according to the pre-specified odd–even rule. Allocation concealment was maintained until the moment of assignment. The study was conducted using a single-blind design in which the outcome assessor was blinded to group allocation.

### Blindness

Due to the inherent differences between the two interventions, blinding of the operating surgeon (RE) was not feasible. However, outcome assessment was performed using a single-blind design. The clinical evaluator (SM) was independent of the treatment procedures and remained unaware of group allocation throughout the study.

#### Preoperative preparation phase

All participants followed the same preoperative protocol to ensure safety and consistency in the procedures. Each participant received a detailed explanation and discussion about the treatment they would undergo. Informed consent was reaffirmed, any remaining questions were answered, and medical histories were reviewed to rule out any contraindications. As part of the preparation, professional dental scaling was performed using an ultrasonic scaler to remove both supragingival and subgingival deposits, which improved oral hygiene before treatment.

#### Operative procedure

The treatment targeted maxillary aesthetic zone with hyperpigmented gingiva, the region extending between the maxillary first premolars. Baseline intraoral photographs were obtained to evaluate pigmentation using the GHP Index. Local anesthetic was administered through infiltration with 2% lidocaine containing epinephrine (1:100,000; Inibsa Artinibsa, Spain) for participants in both treatment groups. 

##### Group I

**Fig. 1:**
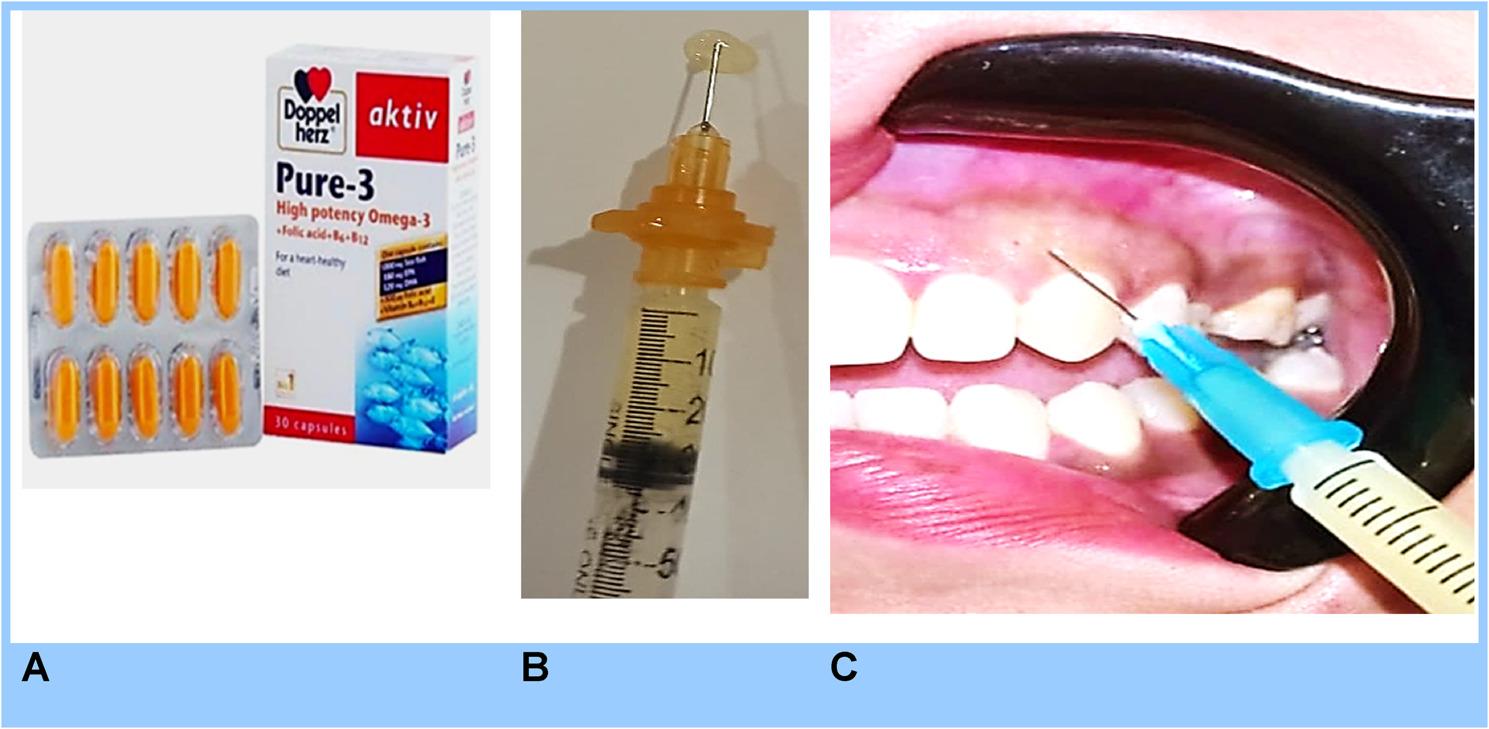
Photograph showing; **A** aktiv Doppel herz^®^ pure-3 high potency omega-3 **B**; after loading the capsule content in the insulin syringe and became ready for injection; **C** Local injection of Omega 3 in the hyperpigmented gingiva

Following local anesthesia, 0.1–0.2 ml of high-potency Omega-3 polyunsaturated fatty acids (1000 mg capsule, Doppelherz, Germany) was used. The capsule contents were emptied into an insulin syringe [26] and then immersed in a warm water bath for 5–10 min to reduce viscosity [27], creating a thinner solution that could be easily injected into the gingival tissue using a 30-gauge, 8-mm insulin needle. Injections were delivered at 2–3 mm intervals along the epithelial–connective tissue interface until visible blanching occurred and were repeated weekly for four consecutive weeks. (Fig. 1**)** In addition, patients received weekly packets of Omega-3 capsules (Provided under the brand name Omega- 3 plus (SEDICO; 6 October City, Egypt) and were instructed to take two capsules daily. The total daily intake of Omega-3 (PUFAs) was maintained at up to 2000 mg throughout the injection period, excluding the day of injection. Compliance was verified at each follow-up by checking returned packets, the study exclude the patients with leftover capsules. Post-injection pain was managed with Diclofenac Sodium twice daily for three days.

##### Group II

**Fig. 2:**
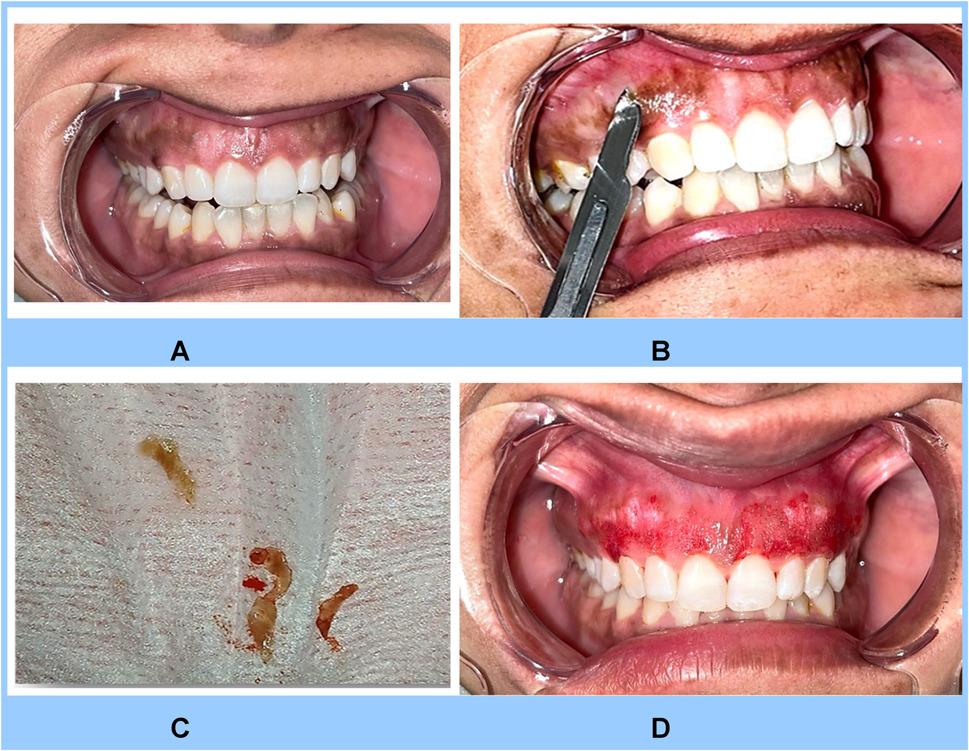
Photograph showing group II procedure; **A** pre-operative photograph for a case with GHP; **B** splitting of hyperpigmented epithelium from the underlying connective tissue; **C** splatted hyperpigmented tissue; **D** immediately after completion of the procedure after bleeding controlled using a pressure pack

Depigmentation was performed with sterile No. 15 surgical blades, gently scraping the pigmented epithelial layer at a 45° angle to preserve the underlying connective tissue (Fig. 2). Hemostasis was achieved using sterile gauze and gentle pressure. No sutures were required, and the treated site was dressed for one hour. Postoperative photographs were taken immediately after the procedure. Prior to surgery, patients were advised to avoid hard, spicy, acidic, and colored foods. Postoperatively, pain was managed with Diclofenac Sodium twice daily for three days.

### Clinical assessment

Comprehensive clinical evaluation was conducted to compare outcomes between the surgical and Omega-3 injection groups. Standardized indices and tools were employed to ensure objective, reproducible, and patient-centered assessment.

A. Dummett Oral Pigmentation Index (DOPI): for measurement of the intensity of pigmentation that was assessed across the six maxillary anterior teeth [28]. This index is based on a four-grade scale (Table 1):
B. Hedin Melanin Index (HMI): [29] for pigmentation distribution which, This index is based on a Five-grade scale (Table 2):

**Table 1:** Scoring criteria dummett index

Score	Grade
0	Gingiva in pink color
1	Mild light brown gingival color
2	Medium or mixed brown & pink gingival color
3	Deep brown or blue-black gingival color

**Table 2:** Scoring criteria hedin index

Score	Grade Description
0	No pigmentation
1	One or two solitary units of pigmentation
2	More than three units of pigmentation in the papillary gingiva without formation of a continuous ribbon
3	One or more short continuous ribbons of pigmentation
4	One continuous ribbon including the entire area between the canines and interdental papilla, with half of the marginal gingiva on either side

Gingival pigmentation indices were estimated at baseline, and at 1-, 3-, and 6-month postoperative time points.

C. Pain: measurement of pain perception was recorded using the Visual Analogue Scale (VAS) [30], a point scale from 0 to 10 where 0 represent No pain, while 10 displayed worst imaginable pain. Participants marked their pain levels immediately post-procedure, on the first postoperative day, and one week later.
D. Gingival Bleeding: recorded post-procedure immediately, bleeding severity was scored using a modified scale [31] (Table 3):

**Table 3:** Bleeding criteria

Score	Description
0	No bleeding
1	Isolated bleeding
2	Mild bleeding
3	Moderate or severe bleeding

E. Wound Healing was evaluated at 1 day and 7–10 days postoperatively using the following scale(12) (Table 4).

**Table 4:** Scoring system for wound healing assessment

Score	Healing Status
0	Presence of necrotic gingival tissue
1	Presence of ulceration in the gingiva
2	Incomplete gingival epithelialization
3	Complete gingival epithelialization

F. Patient Satisfaction and Acceptability [32]: Structured interviews and feedback forms were used to assess patient-reported outcomes at 6-months post-treatment. Parameters included:

**Table:** 

Q1. Did you notice a cosmetic change?			
No		Moderate	marked
Q2. Did the treatment meet your expectation?			
No		Yes	Over and above
Q3. Would you repeat the treatment if necessary?			
Yes	No		

These qualitative insights added to the clinical data and provided a complete picture of how effective the treatment was.

### Statistical analysis

SPSS for Windows, version 26.0, was used to calculate, organize, and evaluate all of the gathered data. The Shapiro-Wilk test assessed the normality of data distribution. The descriptive data was submitted using mean values and standard deviations (mean ± SD). Because the data were non-parametric, the Mann-Whitney U test was used for intergroup comparison. Each group’s time-based changes were examined using the Kruskal-Wallis test. While the Chi-square test was applied to evaluate categorical variables. Statistical significance was determined for all analyses with p-values less than 0.05.

## Result

In the current study, two different depigmentation techniques were investigated on 30 patients allocated to two groups: local injection of omega-3 PUFAs and the scalpel technique. Both groups showed no dropouts or postoperative complications with no adverse reactions were observed among patients who received local injections.

### Demographic characteristics

Group I consisted of 15 participants, including 3 males (20.0%) and 12 females (80.0%), while Group II comprised 15 participants, with 4 males (26.7%) and 11 females (73.3%). The gender distribution of the two groups did not differ significantly, according to a chi-square test, *p* = 0.665. However, within-group analysis indicated a significant gender imbalance in Group I, *p* = 0.020, with a predominance of female participants. In contrast, the gender distribution in Group II did not display statistical significance, *p* = 0.071 (Table 5).

Group I participants range in age range from 22 to 41 years, with a mean age of 26.81 years. Group II had a mean age of 28.53 years, ranging from 22 to 40 years with no statistically significant difference between the groups, *p* = 0.583 (Table 5).

**Table 5:** Displays the demographic data of participants in each group

	Group I	Group II	*P* value
Gender			
Male N (%)	3 (20%)	4 (26.7%)	**0.665 ns**
Female N (%)	12 (80%)	11(73.3%)	
*P* value	0.020**	0.071 ns	
Age			
Mean	26.81 ± 7.47	28.53 ± 6.4	**0.583 ns**
Min-Max	22–41	22–40	

ns Statistically non significant

**= Statistically significant

### Gingival pigmentation intensity

**Fig. 3:**
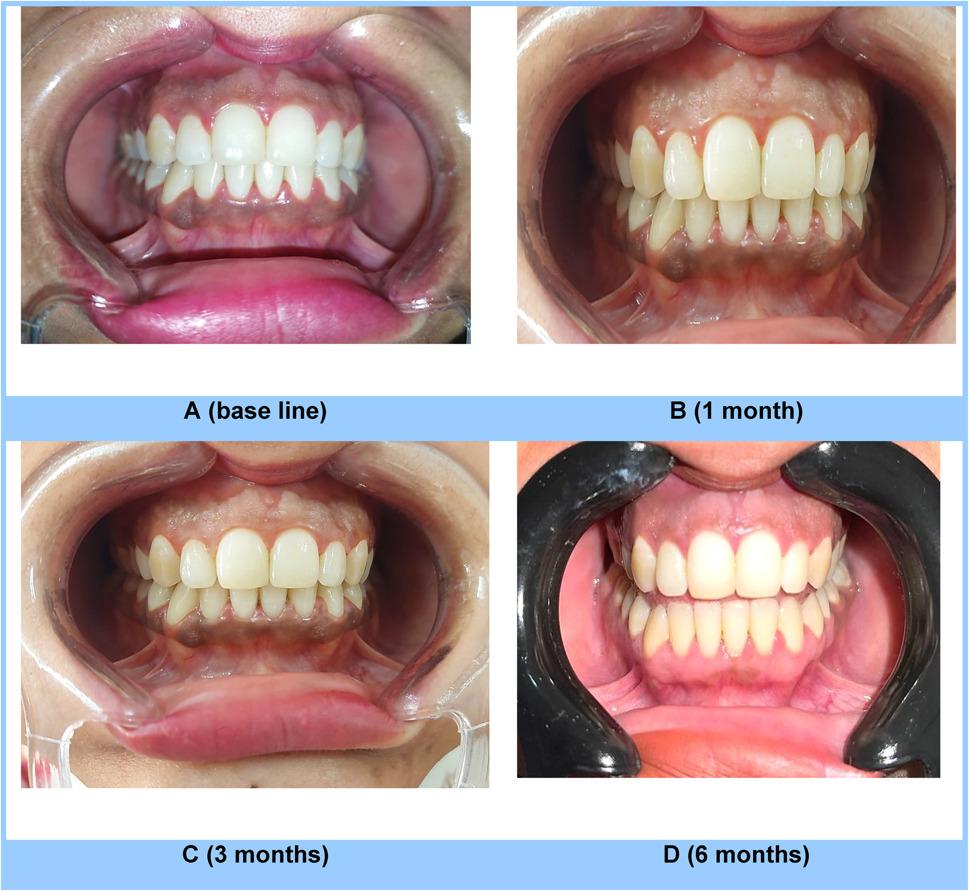
Intra oral images of a patient from Group I at base line (**A**), 1month (**B**), 3months (**C**) and 6months (**D**) after completion of GHP using omega-3(PUFAs) at different time point of follow up

**Fig. 4:**
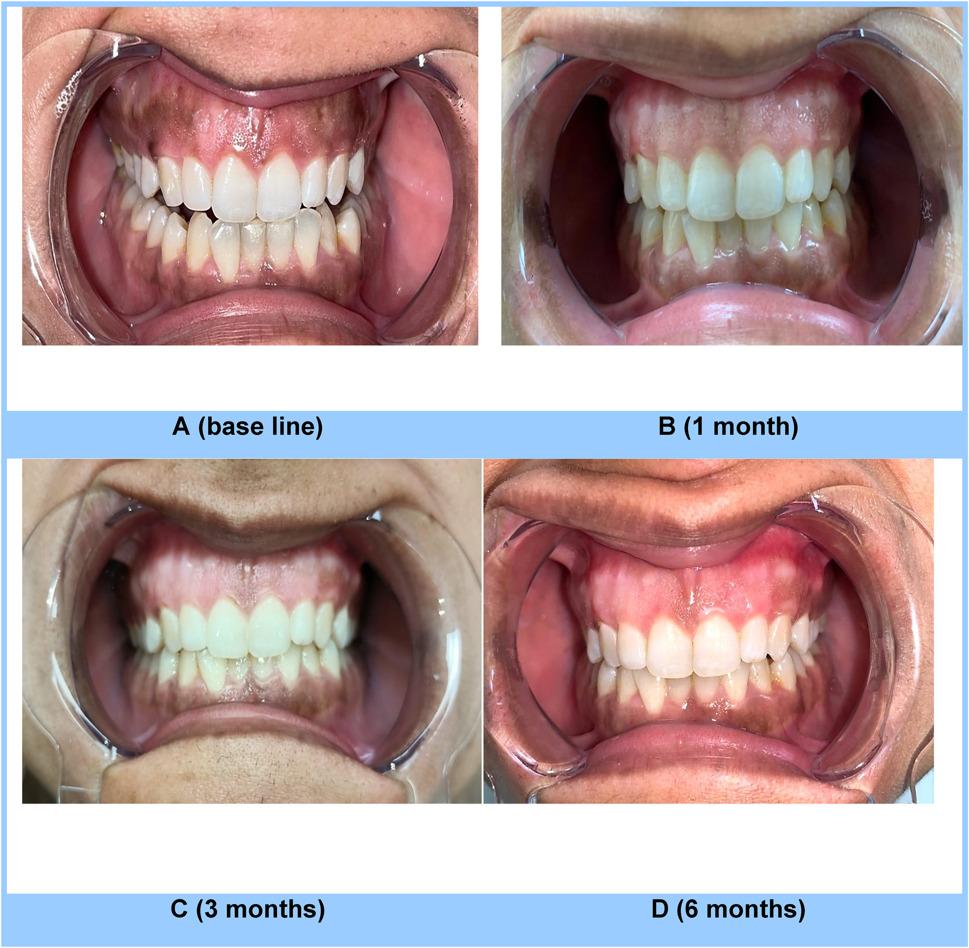
Intra oral images of a patient from Group II at base line (**A**), 1month (**B**), 3months (**C**) and 6months (**D**) after completion of treatment of GHP using surgical lancet blade no 15 at different time point of follow up

From baseline to later time points, both groups represent statistically significant decreases in pigmentation (*p* < 0.001). While the inter-group comparison displayed no statistically significant difference was observed between the groups at baseline (*p ≥* 0.05). However, the pigmentation intensity significantly decreased (*p* < 0.001) in Group II at 1 and 3 months compared to Group I while at 6 months, the difference was no longer statistically significant (*p ≥* 0.05), indicating convergence in pigmentation levels. (Table 6; Figs. 3 and 4)

**Table 6:** Inter-group and intragroup comparison of dummett pigmentation intensity

Time Point DOPI	Group I	Group II	*P*- Value
	M ± SD (MD)	M ± SD (MD)	
Baseline	1.93 ± 0.70 (2) a	2.13 ± 0.74 (2) a	0.455 ns
1 Month	1.27 ± 0.70 (1) b	0.27 ± 0.46 (0) b	< 0.001**
3 Months	0.93 ± 0.70 (1) c	0.27 ± 0.46 (0) b	0.005**
6 Months	0.53 ± 0.52 (1) c	0.60 ± 0.74 (0) b	0.776 ns
P- Value	< 0.001**	< 0.001**	

Different letter indicates significance between intervals within each group

*M* mean, *SD* standard deviation, *MD* median

*p* < 0.05 and *p* < 0.01 indicate statistical significance

ns Statistically non significant

### Hedin melanin index (HMI)

The intragroup analysis revealed a significant difference at each time point when compared to the baseline. However, no statistically significant difference (*p* ≥ 0.05) was noted between the two groups at baseline. Group II exhibited a markedly lower mean pigmentation distribution (*p* ≤ 0.05) at 1 and 3 months compared to Group I. However, By 6 months, there was no longer a statistically significant difference between the groups (*p* ≥ 0.05)., suggesting convergence in pigmentation distribution. (Table 7; Figs. 3 and 4)

**Table 7:** Inter-Group Comparison of Pigmentation Distribution (“HMI”)

Time Point HMI	Group I	Group II	*P* -Value
	M ± SD (MD)	M ± SD (MD)	
Baseline	2.27 ± 0.8 (2) a	2.33 ± 0.98 (2) a	**0.97 ns**
1 Month	1.13 ± 0.64 (1) ab	0.27 ± 0.46 (0) b	0.001**
3 Months	1.07 ± 0.88 (1) b	0.47 ± 0.52 (0) b	**0.07 ns**
6 Months	0.73 ± 0.7 (1) b	0.87 ± 0.74 (1) b	**0.65 ns**
*P*- Value	0.002 **	< 0.001**	

ns Statistically non-significant

**= Statistically significant

### Pain assessment (VAS Scores)

Pain levels were evaluated using VAS at three times: immediately following the procedure, one day post-procedure, and one-week post-procedure. Group I demonstrated a statistically significant (*p* < 0.001) reduction in VAS scores relative to Group II across all time points (Table 4). Within groups, VAS scores also decreased (*p* < 0.001) at one week compared to immediately post-procedure. (Table 8)

**Table 8:** Inter-group comparison of pain (VAS Scores)

Pain (VAS)	Group I	Group II	*P*-value
	M ± SD (MD)	M ± SD (MD)	
after procedure	1.26 ± 0.46 (1) a	2.93 ± 1.43 (3)a	0.0001**
A day	0.67 ± 0.61 (1) b	3.86 ± 1.81 (4) a	0.0001**
a week	0.000 (0) c	1.2 ± 0.56 (0) b	0.000**
P value	0.000**	0.000**	

*VAS* Visual Analogue Scale

**= Statistically significant

### Severity of bleeding

Bleeding severity was assessed using both categorical scores and mean values across Group I and Group II. The findings are summarized in (Table 9).

#### Categorical distribution of bleeding severity

Group I mainly showed mild bleeding, whereas group II exhibited significantly more severe bleeding. The difference in bleeding severity between the groups was statistically significant (χ² test, *p* < 0.001) (Table 9).

**Table 9:** Categorical distribution of bleeding severity

	Group I	Group II	Chi square	*P* value
Scores	N (%)	N (%)	27	< 0.001**
0	1(6.7)	0 (0)		
1	13 (86.7)	0 (0)		
2	1 (6.7)	3 (20)		
3	0 (0)	12 (80)		

Chi-square test used for categorical comparison

**= Statistically Significant

### Wound healing

Wound healing outcomes were assessed at two distinct time points: Day 1 post-procedure and Days 7 post-procedure. Both categorical scores and mean values were analyzed to compare healing progression between Group I and Group II. The findings are summarized in (Table 10).

**Table 10:** Categorical wound healing scores

		Group I	Group II	*P* value
after a day of procedure	Scores	N (%)	N (%)	< 0.001**
	0	0 (0)	1 (6.7)	
	1	0 (0)	5 (33.3)	
	2	2 (13.3)	9 (60)	
	3	13 (86.7)	0 (0)	
7 days of procedures	0	0 (0)	0 (0)	0.03*
	1	0 (0)	0 (0)	
	2	0 (0)	4 (26.7)	
	3	15 (100)	11 (86.7)	
*P* value		0.14	0.000	

#### Categorical distribution of wound healing

On Day 1 and 7 Group I showed markedly statistically significant difference (*p* < 0.05) in wound healing relative to Group II which had lower healing scores. Within groups group I showed no statistically significant difference (*p* ≥ 0.05) across time point in contrast group II displayed statistically significant (*p* < 0.001) improvement at 7 days after the procedure.

### Patient satisfaction

**Fig. 5:**
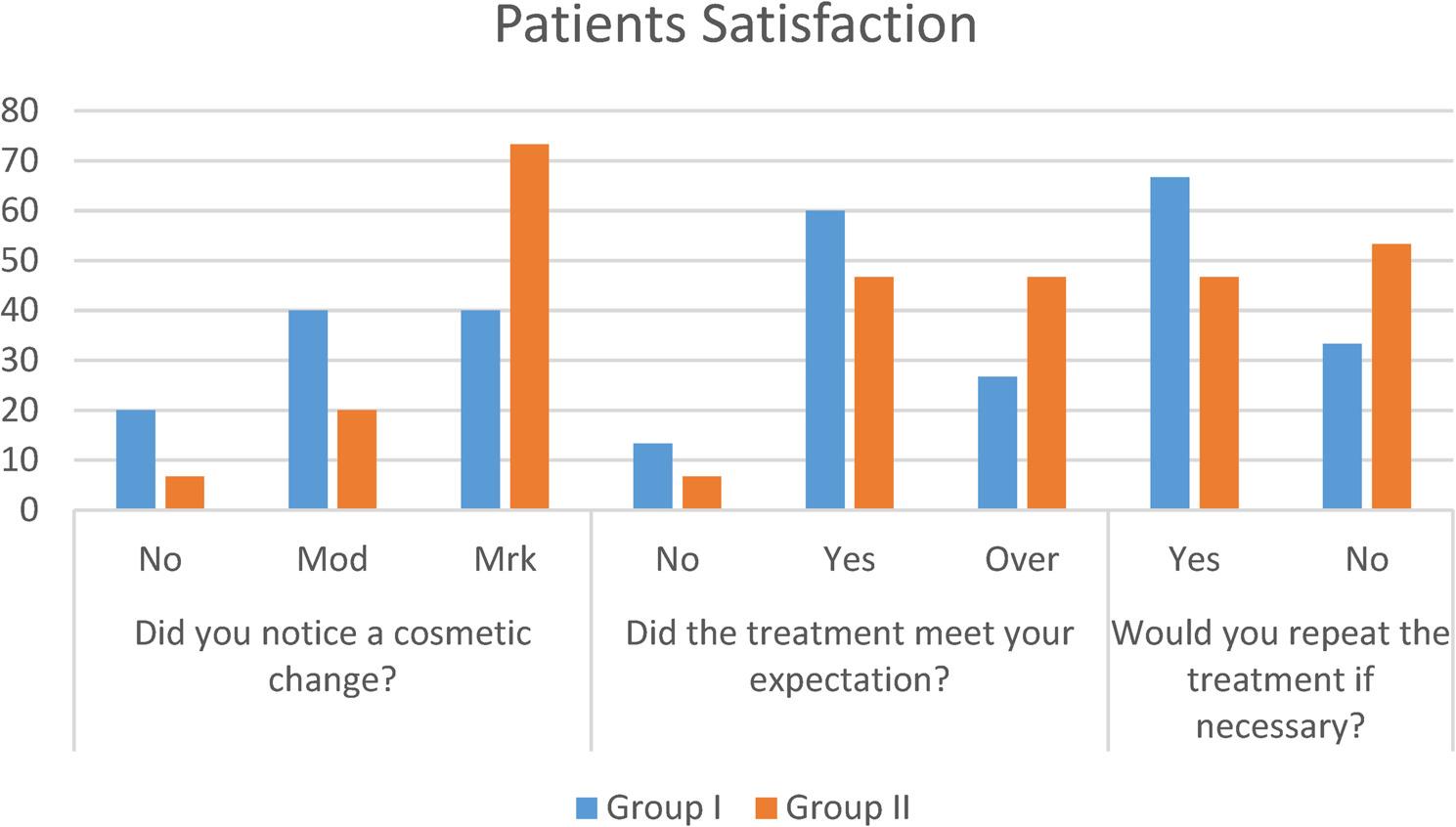
Chart show patient satisfaction graph

Questionnaire for patient satisfaction and acceptability. The study’s findings found no significant differences in patients’ satisfaction and acceptance of the treatment approach between the two groups, as stated in (Fig. 5 and Table 11).

**Table 11:** Showed a comparison of Group I and Group II for each question

Did you notice a cosmetic change?						
	Group I		Group II		Chi square	*P* value
	*N*	%	*N*	%		
No	3	20	1	6.7	3.470	0.1763 ns
Moderate	6	40	3	20.0		
Marked	6	40	11	73.3		
*P* value	0.548 ns		0.003**			
Did the treatment meet your expectation?						
No	2	13.3	1	6.7	1.40	0.496 ns
Yes	9	60.0	7	46.7		
Over and above	4	26.7	7	46.7		
*P* value	0.0742 ns		0.090 ns			
Would you repeat the treatment if necessary?						
Yes	10	66.7	7	46.7	1.221	0.269 ns
No	5	33.3	8	53.3		
*P* value	0.196 ns		0.796 ns			

ns Statistically non-significant

**= Statistically Significant

## Discussion

In the present study, we investigated a combined approach for the treatment of gingival hyperpigmentation (GHP), utilizing local injections of omega-3 polyunsaturated fatty acids (PUFAs) alongside short-term systemic supplementation. The protocol involved four weekly local injections of an omega-3 preparation (0.1–0.2 mg/mL), derived from commercially available oral capsules. The physicochemical properties of PUFAs, specifically their low melting point and liquid state at room temperature, facilitated easy handling and administration [27]. A brief warming period (5–10 min in a water bath) was sufficient to prepare the solution for injection. The preparation also exhibits a near-neutral to weakly acidic pH compatible with soft tissues. Furthermore, the inherent bicarbonate and phosphate buffering capacity of the oral mucosa and gingival tissues helps limit local pH fluctuations and may support tissue homeostasis following localized administration of this lipid-based preparation [33]. 

All procedures were performed under strict aseptic conditions using sterile disposable syringes and needles, and the capsule contents were prepared immediately prior to injection to minimize contamination risk. Throughout the follow-up period, participants were closely monitored, and no clinical signs of infection, tissue necrosis, hypersensitivity, or other local or systemic adverse events were observed, suggesting acceptable short-term biocompatibility. This is consistent with the established topical safety of omega-3 PUFAs, particularly docosahexaenoic acid (DHA) and Eicosapentaenoic acid (EPA), where no cytotoxicity or local reactions have been reported in the literature [34] .

The biological rationale for this approach is supported by a growing body of evidence on the role of omega-3 PUFAs in melanin regulation. Long-chain n-3 PUFAs are integral to cell membrane structure and function, and they can act as signaling molecules, influencing gene expression [35]. Notably, Docosatrienoic acid (DTA) has been shown to inhibit the nuclear translocation of the microphthalmia-associated transcription factor (MITF). MITF is a master regulator of melanogenesis, governing the expression of key enzymes such as tyrosinase, tyrosinase-related protein 1 (TRP-1), and tyrosinase-related protein 2 (TRP-2) [36]. By suppressing this pathway, DTA can lead to a significant reduction in melanin synthesis. This mechanism is further supported by studies on EPA, which have demonstrated its capacity to reduce melanogenesis by downregulating tyrosinase activity and DOPAchrome, without affecting the cellular uptake of tyrosine, the melanin precursor [37]. Our clinical findings align with these cellular mechanisms, as we observed a gradual and consistent decrease in pigmentation intensity and distribution over time. The absence of a significant difference between the 3-month and 6-month follow-ups suggests a therapeutic plateau, indicating that the maximum clinical benefit may be achieved by three months and can be maintained thereafter.

The contribution of the systemic component to the overall outcome warrants careful consideration. While the concurrent daily oral supplementation with 2 g of EPA and DHA (except on injection days) was prescribed to address potential systemic contributors to pigmentation and to synergize with the local therapy, its specific effect is difficult to isolate in the current study design. The four-week duration of supplementation is relatively short, and the observed improvements are therefore most likely attributable to the repeated local injections. Although the FDA generally recognizes up to 3 g/day of marine-sourced omega-3 fatty acids as safe [38], the systemic route in this context was intended as an adjunct. Its precise role in modulating gingival melanin synthesis remains unclear and requires further investigation.

It is also important to acknowledge that part of the initial depigmentation observed may be attributable to the mechanical trauma from the injection procedure itself. The repeated needle penetration could have disrupted the superficial epithelial layer, leading to the removal of melanin-laden keratinocytes and contributing to transient improvement. However, the sustained clinical effect observed over six months suggests that mechanical disruption is not the sole mechanism. The durable result is more plausibly explained by the ongoing biochemical inhibition of melanogenesis by the injected omega-3 PUFAs, as described above.

Our findings with omega-3 PUFAs are comparable to those reported for other mesotherapeutic agents for GHP. Recent studies have demonstrated that locally injected vitamin C is as effective as conventional surgical depigmentation, offering comparable outcomes with the added benefits of better patient tolerability and reduced pain [39, 40]. Similarly, our results indicate that the omega-3 mesotherapy technique produced clinical outcomes comparable to the gold-standard surgical approach. This parity in efficacy was reflected in the patient-reported outcomes; while minor differences in cosmetic results were noted at three months, overall patient satisfaction and treatment acceptability were high in both groups, with no statistically significant differences between them at the six-month follow-up.

This study has several limitations that must be considered when interpreting the results. The most significant is the off-label use of an omega-3 PUFA preparation formulated for oral, not parenteral, administration. Although omega-3 fatty acids are molecules with a favorable biological tolerance, the absence of a certified, pharmaceutically manufactured injectable formulation remains a key limitation. Future research should prioritize the use of such formulations to ensure regulatory compliance and absolute safety.

Second, the study design, which combined local and systemic administration, lacked a systemic-only control group. This prevents a definitive conclusion regarding the independent contribution of oral supplementation. While the four-week systemic regimen was likely too brief to be the primary driver of the sustained six-month improvement, its potential synergistic or adjunctive effect cannot be ruled out. Future randomized controlled trials should include distinct arms for local injection only, systemic supplementation only, and a combination group to precisely delineate the effect of each intervention.

Finally, as a proof-of-concept investigation, this study was designed to assess feasibility, safety, and preliminary efficacy. The six-month follow-up period, while adequate for initial observation, is insufficient to evaluate long-term stability and recurrence rates. Furthermore, clinical observations would be strengthened by correlative histological and molecular analyses to confirm the proposed mechanisms of action within the gingival tissue.

## Conclusion

Within the limitations of this proof-of-concept study, the treatment of gingival hyperpigmentation using a minimally invasive mesotherapy technique with omega-3 polyunsaturated fatty acids, in conjunction with short-term systemic supplementation, produced clinical outcomes comparable to those of the conventional surgical approach. Patient satisfaction was high for both treatment modalities. These findings warrant further investigation through well-designed clinical trials utilizing approved parenteral formulations to establish definitive safety and efficacy. Future research should also focus on longer follow-up periods to assess recurrence and incorporate mechanistic studies to fully elucidate the pathways by which omega-3 PUFAs modulate melanin synthesis in the oral mucosa.

## Data Availability

The datasets used and/or analyzed during the current study are available from the corresponding author upon reasonable request.

## Abbreviations

AA: Arachidonic Acid
ALA: Alpha-linolenic Acid
B16F10: Cell Tumor Factor
B16F10: Cell Tumor Factor
DHA: Docosahexaenoic Acid
DOPA: Dihydroxyphenylalanine
DOPI: Dummett Oral Pigmentation Index
DPA: Docosapentaenoic Acid
DTA: Docosatrienoic acid
EPA: Eicosapentaenoic Acid
EPA: Eicosapentaenoic Acid
GHP: Gingival hyperpigmentation
LA: linolenic Acid
LTB4: Leukotriene B4
LTB5: Leukotriene B5
MITF: Melanocyte Inducing Transcription Factor
n-3: Omega 3
PGE2: Prostaglandin E2
PUFAs: Polyunsaturated fatty acids
SD: Standard deviation
TRP: Tyrosinase Related Protein
TYR: Tyrosinase
VAS: Visual analog scale
α-MSH: Alpha Melanocyte Stimulating Hormone
